# Immobilized Acylase PvdQ Reduces *Pseudomonas aeruginosa* Biofilm Formation on PDMS Silicone

**DOI:** 10.3389/fchem.2020.00054

**Published:** 2020-02-05

**Authors:** Jan Vogel, Marijke Wakker-Havinga, Rita Setroikromo, Wim J. Quax

**Affiliations:** Chemical and Pharmaceutical Biology Department, University of Groningen, Groningen, Netherlands

**Keywords:** N-Acylhomoserine lactones, acylases, quorum sensing, quorum quenching, surface coating, biofilm

## Abstract

The bacterial biofilm plays a key role in nosocomial infections, especially those related to medical devices in sustained contact with patients. The active dispersion of bacterial cells out of biofilms acts as a reservoir for infectious diseases. The formation of such biofilms is a highly complex process, which is coordinated by many regulatory mechanisms of the pathogen including quorum sensing (QS). Many bacteria coordinate the expression of key virulence factors dependent on their population density through QS. The inhibition of this system is called quorum quenching (QQ). Thus, preventing the development of biofilms is considered a promising approach to prevent the development of hard to treat infections. Enzymatic QQ is the concept of interfering with the QS system of bacteria outside the cell. PvdQ is an acylase with an N-terminal nucleophile (Ntn-hydrolase) that is a part of the pyoverdine gene cluster (*pvd*). It is able to cleave irreversibly the amide bond of long chain N-acyl homoserine lactones (AHL) rendering them inactive. Long chain AHLs are the main signaling molecule in the QS system of the gram-negative pathogen *Pseudomonas aeruginosa* PA01, which is known for surface-associated biofilms on indwelling catheters and is also the cause of catheter-associated urinary tract infections. Furthermore, PA01 is a well characterized pathogen with respect to QS as well as QQ. In this study, we immobilized the acylase PvdQ on polydimethylsiloxane silicone (PDMS), creating a surface with quorum quenching properties. The goal is to control infections by minimizing the colonization of indwelling medical devices such as urinary catheters or intravascular catheters. The enzyme activity was confirmed by testing the degradation of the main auto-inducer that mediates QS in *P. aeruginosa*. In this article we report for the first time a successful immobilization of the quorum quenching acylase PvdQ on PDMS silicone. We could show that immobilized PvdQ retained its activity after the coating procedure and showed a 6-fold reduction of the auto-inducer 3-oxo-C12 in a biosensor setup. Further we report significant reduction of a *P. aeruginosa* PA01 biofilm on a coated PDMS surface compared to the same untreated material.

## Introduction

The bacterial colonization of surfaces is an omnipresent observation in almost every environment. However, in healthcare settings biofilms formed by pathogenic bacteria represent a constant threat to the patient's health (Fux et al., 2005). Hospitals follow strict hygiene protocols to keep the clinical environment free of bacteria, however reports show that pathogens can persist for months on surfaces (Kramer et al., 2006). Especially surgical equipment and surfaces need to be sanitized and sterilized, as for example can be seen from the extremely persistent pathogen *Acinetobacter baumannii*. It could be shown that surface attached *Acinetobacter* could withstand dehydration and surface sanitization by ethanol (Harding et al., 2017). Together with *Pseudomonas aeruginosa* this pathogen is a cause for ventilator associated infections (Sievert et al., 2013).

Biofilms can be found on almost every surface ranging from abiotic surfaces to living tissues, such as skin wounds, the lungs and also the teeth. Especially surfaces of medical implants and indwelling medical devices are of importance. In hospital settings indwelling medical devices such as catheters or infusion catheters provide an entryway to the human body making it vulnerable toward bacterial infections (Jacobsen et al., 2008). Since a long time research is being performed to improve urinary catheters especially in respect to minimizing the infection risk by bacteria leading to a catheter-associated urinary tract infections (Lawrence and Turner, 2005). Recently research efforts are being undertaken to reduce the biofilm associated infections by modifying the silicone surface (Swartjes et al., 2013; Ivanova et al., 2015b).

Bacteria organized in a biofilm have been shown to be more resistant to treatment with antibiotics as well as to the host immune system when compared to planktonic pathogens (Ciofu and Tolker-Nielsen, 2010). This has several reasons with one being the protective effect of extracellular polymeric substances and another being the metabolic state of cells persisting within the biofilm. Those cells resemble more the state of cells in the stationary phase (Spoering and Lewis, 2001). In combination with the fact that horizontal gene transfer is promoted within biofilms this creates favorable conditions for the development of resistance of the bacteria (Flemming et al., 2016).

Facing this rise of multidrug resistant pathogenic (MDR) bacteria alternative treatments to antibiotics can be considered to be an effective strategy (Grandclément et al., 2015; Utari et al., 2018). This is generally the case if the patients have to undertake a long term treatment. Accordingly, limiting the formation of biofilms can be beneficial to actively make pathogens more susceptible to antibiotics and also accessible to the host immune response (Brackman et al., 2011; Christensen et al., 2012).

Bacteria can monitor their population density via quorum sensing. Individual cells excrete signaling molecules, so called auto-inducers (Camilli and Bassler, 2006). These signals can be recognized by specialized response regulators which act as transcription factors and play a role in the regulation of various target genes. The auto-inducers do not resemble a single class of molecules but are a diverse group. They differ from gram positive to gram negative bacteria and also within this major groups there are distinguishable differences. In the case of gram negative bacteria the auto-inducing molecules belong to the class of *N-*acylhomoserine lactones (AHL). AHLs consist of a homoserine ring and an acyl chain that can vary in length (Bassler and Losick, 2006). For many pathogenic bacteria, it could be shown that the expression of virulence factors is controlled in a population density depended manner via QS systems (Christiaen et al., 2014). Amongst these virulence factors are also regulative steps toward biofilm formation and differentiation of bacterial biofilms (Fux et al., 2005).

Data collected in the United States suggests that 7.5% of all general healthcare associated infections are connected to *P. aeruginosa* (Sievert et al., 2013). *P. aeruginosa* is a gram negative opportunistic pathogen, which is also considered a model organism for bacterial biofilms. The formation of differentiated biofilm is a complex process containing a number of regulatory networks. However, it could be shown that the *lasIR* QS circuit plays a major role in the development of a mature biofilm. The *lasI* synthase produces N-3-oxo dodecanoyl-homoserine lactone (3-oxo-C12 HSL) (Hentzer et al., 2002).

**Figure 1:**
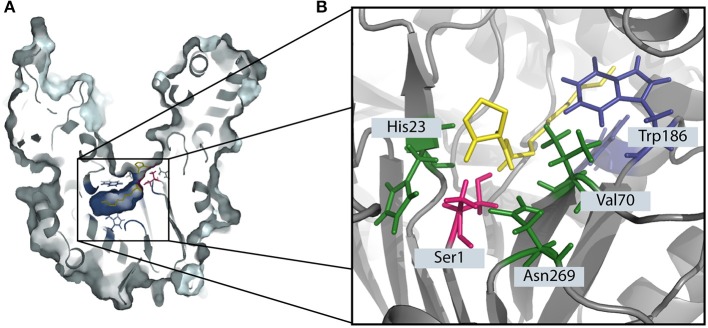
The degradation of 3-oxo-C12 HSL by the Ntn-hydrolase PvdQ. **(A)** The crystal structure of PvdQ (PDB ID: 2WYC) shows the orientation 3-oxo-C12 HSL in the hydrophobic binding pocket (indicated in blue). The N-terminal amino acid serine (highlighted in pink) launches a nucleophilic attack on the amid bond of the auto-inducer. **(B)** The amino acids Val70, His23, and Asn269 in the active center stabilize the transition state, forming the oxyanion hole. Hydrophobic AA such as Trp186 form a hydrophobic pocket.

PvdQ is a Ntn-hydolase from *P. aeruginosa* PAO1, which is studied as a QQ enzyme aimed at degrading long chain AHLs of gram negative pathogens. All Ntn-Hydrolases share a similar hydrolysis mechanism. The N-terminal amino acid, which can be a serine, cysteine or a threonine launches a nucleophilic attack on the carbonyl carbon of the substrate. This results in a tetrahedral intermediate which is stabilized by residues in the active center (Figure 1B) (Peräkyl and Rouvinen, 1996). Subsequently, the α-amino group of the nucleophile donates its proton to the nitrogen of the scissile peptide bond. This results in the formation of a covalent bond with a part of the substrate, while the leaving amino product is released. In a following deacetylation step the carbonyl group of the acyl-enzyme complex will be cleaved in the presence of water (Duggleby et al., 1995). In the case of PvdQ it could be shown that the acyl group of the AHL perfectly fits into a hydrophobic pocket at the active centre (Figure 1A), placing the amide bond in proximity to the N-terminal nucleophile (Bokhove et al., 2010). It could be successfully utilized to reduce the virulence of *P. aeruginosa* PAO1 in several infection models such as *Caenorhabditis elegans* and a mouse infection model (Papaioannou et al., 2009; Utari et al., 2018).

**Figure 2:**
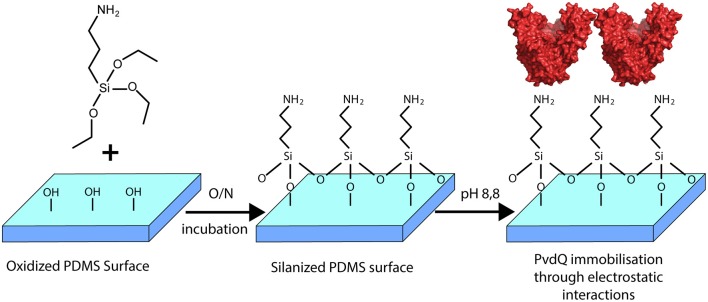
Overview of the surface modification with PvdQ. PDMS silicone surface is treated with (APTES) introducing free amid bonds to the surface. In the chosen pH conditions, due to the negative charge of PvdQ, the protein attaches electrostatically to the surface.

In this work we constructed a surface with QQ properties utilizing the quenching potential of the acylase PvdQ. The surface of interest in this study is PDMS, which is extensively used to make urinary catheters. Many groups worldwide are working on surface modifications for this material (Diaz Blanco et al., 2014; Francesko et al., 2016). This is the first time a successful immobilization of the acylase PvdQ derived from *P. aeruginosa* was performed. The chosen immobilization technique is based on electrostatically interactions and thus makes it possible to work under mild conditions for the protein (Figure 2) (Ivanova et al., 2015a). Furthermore we present proof of enzyme attachment to the surface as well as confirm its activity with a biosensor based activity assay. The functionalized surface shows a significantly lower level of colonialization than compared to untreated PDMS. This strategy may be used to degrade the QS signaling pathway of gram-negative bacteria to interfere with the biofilm formation on PDMS surfaces.

## Materials and Methods

### Bacterial Strains and Growing Condition

*P. aeruginosa* PAO1 was obtained from Barbara Iglewski (University of Rochester Medical Center, Rochester, NY) (Sio et al., 2006). The overnight cultures of the biosensors were prepared by inoculating a loop of frozen glycerol stock in Luria Bertani (LB) medium, followed by incubation at 37°C, 200 rpm. *E. coli* JM109 pSB1142 was inoculated in LB and incubated at 37°C, 200 rpm with selective AB supplementation (Winson et al., 1998).

### PDMS Silicone

The polydimethylsiloxane was prepared using a Sylgard 184 elastomer kit acquired from Dow Corning, VWR chemicals, Amsterdam, The Netherlands, and the preparation was done according to the supplier's information. PDMS samples were prepared by mixing the silicone elastomer base and silicone curing agent at a weight ratio of 10:1. To prevent bubble formation, the mixture was degassed under vacuum, providing a 1 mm thick PDMS substrate. Molds were placed in an oven and cured at 70°C overnight. The surface roughness of the PDMS was measured using atomic force microscopy (AFM) model Dimension 3100 Nanoscope V system (Veeco, Plainview, NY, USA) in contact mode and with 0.24 N/m tips. All data were processed using Nanoscope Analysis (Veeco, Version 1.70). The surface roughness was calculated on a 5 × 5μm^2^ region. The mean surface roughness (Ra) was 3 nm.

### Activity Assay

The enzymatic degradation of long chain AHLs was measured by employing the biosensor strain *E. coli JM109* carrying the reporter plasmid pSB1142 (Winson et al., 1998) The vector is containing a *lux* operon, which is under control of a *lasI* promotor. The corresponding transcription factor is the quorum sensing response regulator LasR. Upon the binding of long chain AHLs ranging from C10 to C14 carbon chains the activated response regulator LasR upregulates the Lux operon and thus detects their presence with light emission. The amount of light produced by the biosensor was measured every 5 min during a 15 h time course in a multifunctional microplate reader (FLUOstar Omega, BMG Labtech). Data points obtained immediately prior to maximum light production were used for comparisons.

The activity of the immobilized enzyme PvdQ was assessed with this biological assay. In brief, PDMS slices of 3 × 15 mm were incubated in 400 μl 50 mM Tris-Cl pH 8.8 containing 0.1 mM 3-oxo-C12 HSL. The PDMS samples were incubated for 24 h at 30°C shaking at 100 rpm. After the incubation 100 μl of the buffer solution was used for the biosensor assay. Measurements were performed three times in individual experiments in quadruplicates.

### PvdQ Purification

PvdQ was produced and purified as reported previously (Bokhove et al., 2010). In short: *E. coli DH10B* carrying the plasmid pMCT_*PvdQ* was grown in 2xTY medium containing 16 g/l Tryptone, 10 g/l Yeast extract, 5 g/l as well as chloramphenicol (50 μg/mL) for 30 h at 30°C, 200 rpm. The cells were harvested and sonicated in a three times volume of lysis buffer (50 mM Tris Cl pH 8.8; 2 mM EDTA). Cell lysate was centrifuged at 17.000 rpm for 1 h. All purification steps were performed with an Äkta Pure system from GE Healthcare Life Sciences. The clear lysate was applied to an anion exchange HiTrap Q-sepharose column, PvdQ can be detected in the flow through. Next purification step is with a hydrophobic interaction phenyl sepharose column, for this the flow through is diluted in buffer with 2.8 M ammonium sulfate concentration to a final concentration of 750 mM. PvdQ could be detected at the end of the ammonium sulfate gradient elution step. Lastly the flow through was applied to a gel filtration superdex75 16/60 column. PvdQ was collected and stored at −80°C until further use. All protein chromatography columns were obtained from GE Healthcare Life Sciences.

### Fluorochrome Labeling of PvdQ

To be able to detect PvdQ on the PDMS surface, 200 μl PvdQ was first labeled with 20 μl FITC thiocyanate (0.1 mg/50 μl DMF) overnight at 4°C according to manufacturer's manual. The unbound FITC was removed with a PD10 Desalting column (GE Healthcare). The labeled protein was incubated together with respective PDMS slices of 5 × 15 mm to confirm the attachment of the protein to the PDMS surface. All experiments were done with the same batch of labeled PvdQ.

### Biofilm Assay

The ability of *P. aeruginosa* strains to attach to surfaces as an initial step of the biofilm formation was tested by using Crystal violet staining. The attached cell mass was determined by a biofilm assay published by O'Tool et al. with modifications (O'Toole, 2010). Briefly, bacterial strains were grown overnight at 37°C and were diluted in LB broth to a concentration of OD 0.8. Aliquots of 100 μl were added to wells of a 96-well plate containing a PDMS piece (3 × 15 mm) and incubated at 37°C for 3 h to allowing the bacteria to adhere to the surface. The medium and planktonic cells were removed and the PDMS slices were washed three times with 200 μl PBS. The Biofilms were fixed for 60 min at 60°C and stained using 150 μl 0.1% (v/v) crystal violet solution for 15 min. The PDMS slices were washed in distilled water, and the water was removed. The plate containing the PDMS slices was then air-dried and the dye was eluted with 110 μl 33% acetic acid. The signal was measured using a FLUOstar Omega plate reader (BMG Labtech) at a wavelength of 585 nm. Measurements were performed in three biological repetitions consisting of 4 replications each. The reduction of cell mass was assessed by comparing treated and untreated samples. As a negative control to assess the amount of background dye which is staining PDMS an additional staining was performed with wells only containing PDMS and medium without bacterial cells. This was considered the technical negative control.

## Results

### Attachment of QQ Acylase PvdQ to PDMS Silicone

**Figure 3:**
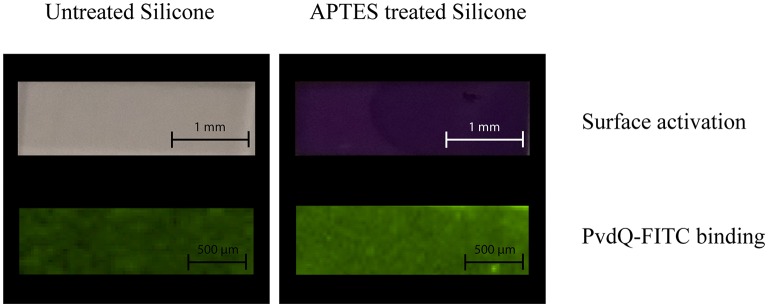
Surface activation and protein binding on treated and untreated PDMS. The upper row shows the surface activation, comparing untreated PDMS (right column) and APTES treated PDMS (left column) both stained with 2% Ninhydrin solution. The lower row shows the attachment of FITC labeled PvdQ on untreated PDMS (left column) and APTES treated PDMS (right column).

As a proof of principle to confirm the activation of the PDMS surface as well as the attachment of the acylase PvdQ to this activated surface we performed the following experiment before proceeding to activity assays. The PDMS was washed and the surface activation was confirmed by immersing the PDMS in a 2% Ninhydrin solution. As shown in Figure 3 an APTES treated PDMS sample incubated in Ninhydrin solution shows a clear deep purple signal confirming the uniform activation of the surface. The comparison to a piece of PDMS which was not pre-treated with APTES acted as a control experiment. Figure 3 clearly shows no color staining and hence indicates that there is no surface modification. After proofing the success of the surface activation, the conditions were used for all the following coating experiments. The negative net charge of the acylase PvdQ under this conditions was confirmed by a measurement of the zeta potential (Table S1). The chosen pH conditions and the resulting negative net charge of PvdQ make it possible to perform a protein immobilization based on electrostatic interactions between surface and enzyme.

Next, we confirmed the binding of FITC labeled PvdQ to the activated PDMS. For this the APTES activated PDMS surface was incubated in a 1.5 mg/ml solution of the acylase PvdQ for 24 h. The chosen buffer conditions were set to a pH of 8.8. To proof a successful attachment of the enzyme, we used FITC labeled PvdQ acylase and incubated it together with pretreated PDMS under storage buffer conditions for 24 h. The immobilized PvdQ acylase on the surface was visualized under UV light. FITC labeled PvdQ could clearly be recognized on the PDMS surface under UV light (Figure 3). The same experiment was performed with non APTES activated PDMS showing a residual luminescence which was significantly less than the PDMS with the APTES activated surface (Figure 3). This experiment clearly confirms the attachment of the acylase PvdQ to the surface.

### Activity of Immobilized QQ Enzyme PvdQ

**Figure 4:**
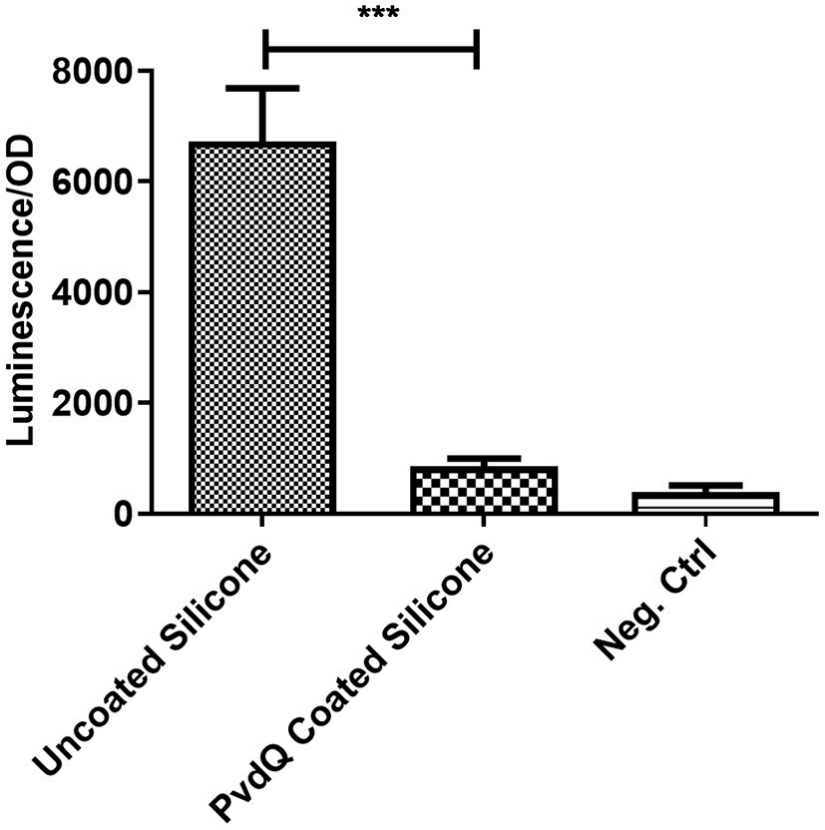
Biosensor based activity assay of immobilized enzyme. The activity of the immobilized enzyme was measured in a biosensor set-up. The readout is a luminescence upon the presence of 3-oxo-C12 HSL, the signal is normalized to the OD of the biosensor. Untreated PDMS shows a strong light emission compared to the PvdQ coated silicone. This means a reduction of 3-oxo-C12 HSL. The negative control emits a weak signal and acts as the baseline of the experiment. Measurements were performed three times in individual experiments (***p < 0.001).

To assess the activity of the immobilized enzyme, the coated PDMS was incubated for 24 h at 30°C in the presence of 3-oxo-C12 HSL. This molecule is the main auto-inducer of the pathogenic bacterium *P. aeruginosa*. Previous studies showed that this signaling molecule is involved in the biofilm formation and hence is the molecule of interest for this study (de Kievit, 2009). In this experiment the presence of AHLs was determined in a biosensor setup. After the incubation time the quenching ability was measured by comparing the AHL levels after incubation in the set-ups with coated and uncoated PDMS slices. In the set-up with the uncoated PDMS (Figure 4) a high luminescence signal of the biosensor system can be observed indicating the presence of AHLs. Compared to the same set-up including the PDMS with the immobilized acylase PvdQ a 6-fold reduction in luminescence is visible, showing that the enzyme is able to degrade the signal molecule and thus shows activity. As a positive control free acylase in solution was used to confirm that the assay shows the right sensitivity to measure QQ activity. This experiment confirms a successful functionalization of a PDMS surface with QQ properties.

### Inhibition of Biofilm Formation

**Figure 5:**
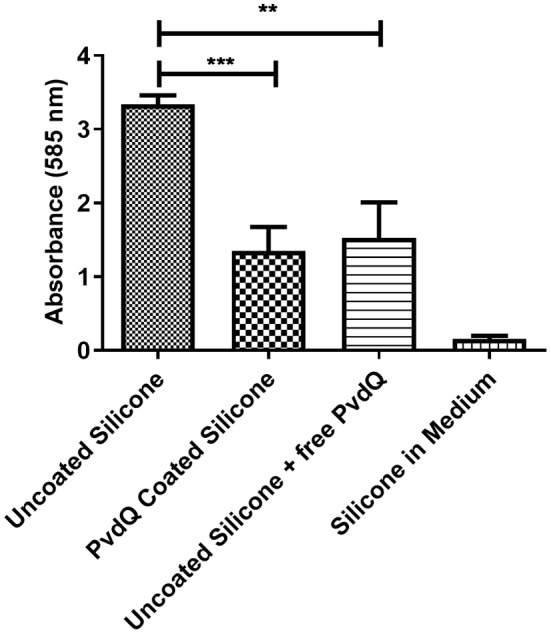
Biofilm formation on coated and uncoated PDMS. The biomass on the PDMS pieces was stained and measured with a 0.1% crystal violet solution. All samples are incubated under the same conditions. The negative control was incubated in sterile medium. The higher signal of the uncoated PDMS pieces compared to the PvdQ coated PDMS shows the reduction in surface attached biomass. Measurements were performed three times in individual experiments. Uncoated PDMS in addition with free acylase in the medium shows a comparable effect (**p ≤ 0.01; ***p ≤ 0.001).

After confirming that the PDMS silicone surface was functionalized with QQ properties we were investigating if the QQ potency of the treated PDMS surface has an impact on the capability of *P. aeruginosa* PAO1 cells to attach to the surface and form a biofilm. For this purpose we grew a biofilm on PDMS slices by submerging coated and uncoated PDMS slices in complex medium inoculated with *P. aeruginosa* PAO1. The cells were given time to attach to the surface and the biomass on the PDMS was measured using the crystal violet staining method. Accordingly, the colonization of treated and untreated PDMS was compared to evaluate the reduction in biofilm formation. It could be observed that the initial attachment of bacterial cells was significantly reduced, compared to untreated silicone (Figure 5). The absorbance signal resembling the biomass on the PDMS slices shows a reduction of about 50% on the treated surface compared to the untreated surface (Figure 5). As a control experiment untreated PDMS in combination with 500 mg/ml QQ soluble acylase PvdQ was used showing a comparable result. This highlights the similarity between the QQ effect of PvdQ in solution, as well as the quenching effect of the enzyme when immobilized on a surface. To ensure that the biomass staining is not a result of attachment of parts of the complex medium to the PDMS surface, we incubated an uncoated PDMS slide in sterile medium. The measurement confirms that the signal measured represents the attached biomass to the surface and it is not due to unspecific binding of crystal violet to the PDMS surface.

## Discussion

Studies have shown that even though the formation of a bacterial biofilm is not solely depending on QS but involves a multitude of different regulatory networks, QS systems play an important role in the formation and differentiation of a mature *P. aeruginosa* biofilm (Stoodley et al., 2002).

In this study we purified the Ntn-hydrolase PvdQ derived from *P. aeruginosa* PAO1 and immobilized it on a silicone surface in a comparable fashion to Ivanova et al. (2015b). One natural function of this enzyme is removing the fatty acid chain from the pyoverdine precursor PVDIq (Nadal-Jimenez et al., 2014). Furthermore, in previous studies we also could show that PvdQ effectively degrades long chain AHLs and has an impact on the virulence of *P. aeruginosa* (Jimenez et al., 2010; Utari et al., 2018).

A first step toward a successful immobilization was to find a surface activation that can be used for the attachment of PvdQ. We performed a surface activation with APTES and we could see a full activation by staining the treated PDMS with a 2% Ninhydrin solution. This confirmed the presence of free amid bonds on the surface. Incubating the treated PDMS in a 1.5 mg/ml solution of FITC labeled PvdQ showed a clear fluorescent signal when viewed under UV light after washing the PDMS slides in distillated water. Based on the UV-Vis measurement of the FITC-conjugated PvdQ we could see that the amount of FITC labeling of the protein was sufficient to observe a clear signal on the PDMS surface. We could see a clear difference between the APTES activated and the untreated PDMS surface as shown in Figure 3. This confirms that PvdQ can be attached via interactions with the APTES activated PDMS surface.

We could show that PvdQ retained its activity when immobilized on the PDMS surface in our experimental conditions. The biosensor set-up showed a 6-fold difference between the sample containing the uncoated PDMS and the sample that harbored the PDMS slice with immobilized PvdQ. The signal reduction represents the quenching of the auto-inducer signal 3-oxo-C12 HSL.

To explore the QQ effect of the PvdQ coated surface we performed a biofilm assay with *P. aeruginosa* PA01. Most importantly we could show in this biofilm set-up a significant reduction of the attached biofilm on the treated PDMS in comparison to the same material without the immobilized acylase. Comparing the immobilized PvdQ with the soluble PvdQ in combination with untreated PDMS, we could see a similar reduction of attached bacterial cell mass. Both of these set- ups showed a reduced biofilm formation compared to the untreated PDMS without PvdQ. The comparison of the PvdQ treated surface and the untreated surface with added PvdQ leads to the conclusion that the reduction in biomass is indeed due to the quenching ability of PvdQ (Figure S1). Ivanova et al. could show in their research that a surface functionalized with a commercially available acylase preparation from *Aspergillus melleus* can reduce the bacterial biofilm (Ivanova et al., 2015b). In this study we utilized the well-characterized acylase PvdQ, which is here shown to have QQ properties due to activity toward long chain AHLs. It has been shown in our group that this enzyme also has a positive impact on the course of a *P. aeruginosa* induced mouse infection model, which shows the potency of this enzyme as a potential treatment option (Utari et al., 2018).

The reduction of biofilm formation on the PDMS surface shows that the acylase PvdQ can have preventive anti biofilm properties by hindering the initial attachment of *P. aeruginosa* cells, which is mediated by degrading the auto-inducer 3-oxo-C12 HSL. Biofilms are highly organized structures and exhibit a complex architecture. This differentiation requires a high degree of regulation with the involvement of the QS system (Chang, 2018). In *P. aeruginosa* it is known that the las system, which is utilizing 3-oxo-C12 as its cognate signaling molecule, plays a crucial role in the differentiation of the biofilm. A deletion of the las systems leads to a biofilm, which is flat and does not show the same differentiated phenotype as the wild type biofilm (Rasamiravaka et al., 2015). Culturing conditions play an integral role of the phenotype of the biofilm (Kirisits and Parsek, 2006). In the case of *P. aeruginosa* biofilms it could be shown that under iron limiting conditions, mutations in iron acquisition genes such as *pvdA* have a huge influence on the biofilm morphology (Banin et al., 2005). In this background it is essential for this work to highlight the necessity to explore different growth conditions.

This knowledge can be applied on various medically relevant surfaces especially of indwelling medical devices. PvdQ specifically targets long chain AHLs such as 3-oxo-C12 HSL, which is the main auto-inducer used by *P. aeruginosa* PAO1. Immobilizing this enzyme on a surface provides a surface that shows a significant reduction in the attached cell mass in the initial stage of the biofilm formation, which is the crucial step toward the development of a mature biofilm. This technique has the potential to contribute to novel strategies to prevent nosocomial infections targeting especially pathogens based on their respective QS system.

Furthermore, utilizing QQ strategies as a potential treatment could prove advantageous since it does not apply direct selective pressure on the pathogens. Accordingly, it is hypothesized that the development of resistance strategies is less likely to occur compared to antibiotic treatments.

Treatment options such as QQ are a promising strategy to overcome problems of rising numbers in multi-drug-resistant bacterial infections. On the surface dispersed bacterial cells from biofilms can act as a reservoir for infection. QQ strategies on medical surfaces can reduce the biofilm load which can result in a lower number of biofilm originated pathogens and thereby support the host immune system. In this work we could show that the attachment of *P. aeruginosa* cells to an abiotic PDMS surface could be significantly reduced in laboratory conditions. The potential of PvdQ as a treatment strategy against bacterial infections could be shown in previous studies in our group in which dissolved PvdQ was administered intranasal to the lungs. We could observe a protective effect of PvdQ against a *P. aeruginosa* induced pulmonary infection in a mouse model. Furthermore the same study pointed out that PvdQ does not show any negative effect in the lung environment of the mouse model and can be regarded as safe (Utari et al., 2018). The results presented here show that PvdQ has a positive effect on the biofilm inhibition, however we have to take into account that different environmental factors play a role on the formation of biofilms (Kirisits and Parsek, 2006). Further research has to be conducted to show the potency of PvdQ as possible treatment option under more clinical conditions.

The QQ strategies we present in this work could be tailor made strategies targeting gram negative pathogens such as *P. aeruginosa* and *A. baumannii*, since these organisms communicate with long chain AHLs. Furthermore previous work in our group demonstrated that an engineered PvdQ variant was able to degrade the auto-inducer used by *Burkholderia cenocepacia* and had an positive effect on an performed infection model (Koch et al., 2014). Enzymatic QQ efforts toward gram positive bacteria are to our knowledge still vastly undiscovered. The only characterized QQ enzyme targeting auto-inducer 2 of gram positive bacteria is the kinase Lask (Roy et al., 2010). However, its dependency on ATP make this enzyme a difficult candidate as a treatment option.

In conclusion the interference with the QS system with QQ enzymes can be described as a promising strategy to fight infectious diseases. We demonstrate a significant reduction of *P. aeruginosa* cell attachment in the early biofilm state. It is very likely that MDR strains are susceptible toward QS interference and since the treatment does not apply direct evolutionary pressure on the pathogens it is hypothesized that a QQ treatment could be a lasting treatment strategy for longer hospitalized patients. After achieving a successful immobilization of the QQ acylase PvdQ future perspectives are the refinement of the coating technique and a conclusive analysis of its stability as well as studying different environmental conditions for the biofilm formation. The findings presented here open up the way toward investigations applying the immobilized PvdQ in an animal model for catheter applications.

## Data Availability Statement

All datasets generated for this study are included in the article/Supplementary Material.

## Author Contributions

WQ was the principal investigator who initiated the project of quorum quenching. JV designed the experiments. MW-H, RS, and JV performed the experiments and analyzed the data. The manuscript was written by JV and was carefully revised by WQ.

### Conflict of Interest

The authors declare that the research was conducted in the absence of any commercial or financial relationships that could be construed as a potential conflict of interest.
