# Supplementary material for: Immobilized Acylase PvdQ Reduces Pseudomonas aeruginosa Biofilm Formation on PDMS Silicone
Source: Front Chem. 2020 Feb 5;8:54. doi: 10.3389/fchem.2020.00054 (PMC7012999; doi:10.3389/fchem.2020.00054)
Supplement: Supplementary file 1 [file Table_1.DOCX]

**Table S1** is showing the measurements of the Zeta potential of the acylase PvdQ.

**Figure S1 Biofilm assay on PvdQ coated silicone and BSA coated Silicone.** The determination of the biomass on the PDMS surface was done with a 0,1% crystal violet solution. All samples are incubated under the same conditions. The negative control was incubated in sterile medium. The comparison between the BSA coated and the PvdQ coated surfaces showed a significant reduction of biomass on the PvdQ PDMS slice. In addition an uncoated PDMS slice with free acylase in the medium showed a comparable effect as the PvdQ coated surface.
