# Supplementary figures and images for: Immobilized Acylase PvdQ Reduces Pseudomonas aeruginosa Biofilm Formation on PDMS Silicone
Source: Front Chem. 2020 Feb 5;8:54. doi: 10.3389/fchem.2020.00054 (PMC7012999; doi:10.3389/fchem.2020.00054)

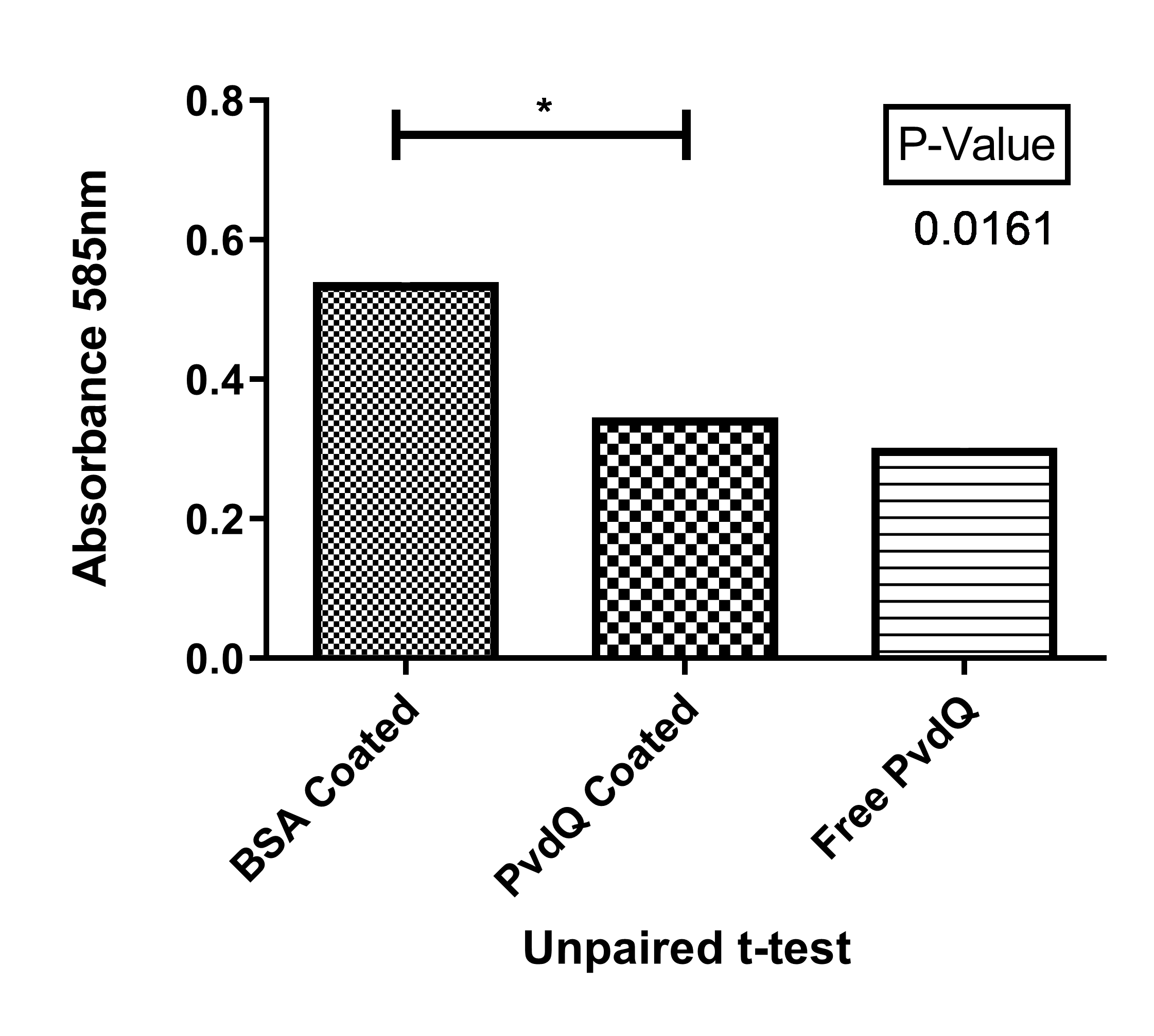

Supplement: Supplementary file 2 [file Image_1.TIF]

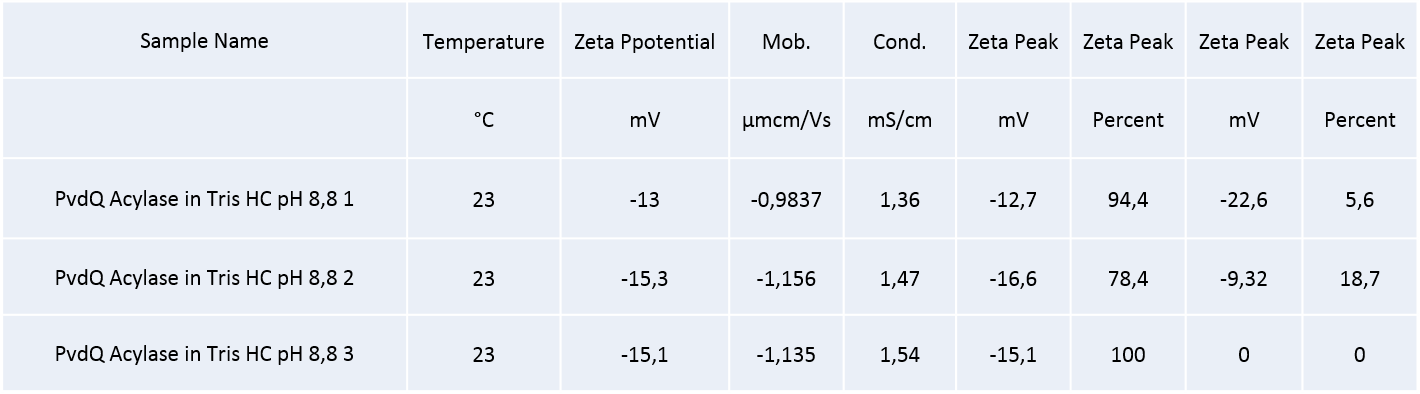

Supplement: Supplementary file 3 [file Image_2.TIF]
